# Alpha-Lipoic Acid in Diabetic Peripheral Neuropathy: Addressing the Challenges and Complexities Surrounding a 70-Year-Old Compound

**DOI:** 10.3390/cimb47060402

**Published:** 2025-05-29

**Authors:** Iliya Mangarov, Yulian Voynikov, Valentina Petkova, Simeon Iliev, Ivanka Kostadinova, Lyubomir Marinov, Irina Nikolova

**Affiliations:** 1 Department of Neonatology, University Hospital “SofiaMed”, Faculty of Medicine, Sofia University, St. Kliment Ohridski, 1504 Sofia, Bulgaria; 2 Department of Chemistry, Faculty of Pharmacy, Medical University, 1000 Sofia, Bulgaria; y_voynikov@pharmfac.mu-sofia.bg; 3 Department of Organization and Economics of Pharmacy, Faculty of Pharmacy, Medical University of Sofia, 1000 Sofia, Bulgaria; 4 Independent Researcher, 1680 Sofia, Bulgaria; 5 Department of Pharmacology, Pharmacotherapy and Toxicology, Faculty of Pharmacy, Medical University of Sofia, 1000 Sofia, Bulgaria

**Keywords:** diabetic peripheral neuropathy, alpha lipoic acid, thioctic acid, diabetic neuropathy management, safety, efficacy, market share

## Abstract

Alpha-lipoic acid (ALA, also known as thioctic acid) was discovered nearly 90 years ago and began to be used in clinical practice in the late 1950s. Numerous nonclinical and clinical studies have investigated ALA for treating diabetic peripheral neuropathy (DPN) and various other diseases. The rising global prevalence of DPN necessitates timely treatment; however, there is currently no effective cure. Current guideline-recommended therapies for DPN provide symptom relief rather than modifying the disease. Among the pathogenesis-oriented therapies, ALA holds a unique position as a universal antioxidant, essential for every cell in the body. This review highlights the ongoing issues and challenges in using ALA to treat DPN. While confronting a complex disease with poorly understood pathophysiology, we also have an endogenous substance with pleiotropic effects on all cells in the human body. It becomes clear that this is a highly multifactorial process that will likely never be precisely defined. This does not diminish the significance of ALA in treating DPN but underscores the need for a deeper understanding of when to start therapy, dosage, duration, and monitoring. In this comprehensive review, we evaluate the achievements of the past 70 years and highlight gaps in ALA’s role in treating DPN.

## 1. Introduction

Alpha-lipoic acid (ALA or thioctic acid) has been extensively studied since its first isolation in 1951 [1,2]. ALA is an essential endogenous substance biosynthesized in mitochondria and exhibits an impressive range of pleiotropic biological effects that undoubtedly influence whole-body physiology [3,4,5,6,7,8,9]. ALA’s specific mechanisms and effects across various pathological conditions are not fully clarified. Its antioxidant properties seem to underlie its beneficial health effects in managing various pathological conditions [2,8,10,11,12,13,14,15]. ALA and its metabolite dihydrolipoic acid (DHLA) are considered “universal antioxidants” and function as biological antioxidants, metal chelators, regenerators of other antioxidants, and modulators of several signaling pathways.

Several pre-clinical in vitro and in vivo studies, randomized, double-blind, placebo-controlled trials, and subsequent systematic reviews and meta-analyses demonstrate ALA’s potential in preventing or delaying the onset of diabetic complications, namely diabetic peripheral neuropathy (DPN) [15,16,17]. DPN is the most prevalent form of neuropathic pain, affecting around 50% of diabetic patients [18,19,20]. It typically manifests as a chronic, symmetrical, length-dependent sensorimotor polyneuropathy and represents a significant cause of non-traumatic amputations [21,22,23]. However, the mechanisms by which diabetes leads to these complications and disease progression remain largely unclear. These processes are complex, stemming from an interplay of various interacting factors. A general problem with all preclinical and clinical studies is the selection of the optimal dose of ALA, treatment duration, trial outcomes, and the specific metabolism of the antioxidant, which results in somewhat contradictory patient outcomes [24].

Structurally, as a sulfur-containing substance, ALA is considered a thiol compound. It exists as two enantiomers: (R)-(+)-lipoic acid (R-ALA) and (S)-(−)-lipoic acid (S-ALA), and as a racemic mixture. Only the R-(+)-enantiomer is synthesized in small amounts by microorganisms, plants, animals, and humans, and it is biologically active. Racemic ALA and its biologically active enantiomer, R-ALA, are marketed globally as both therapeutic agents and nutritional supplements in various dosage forms ranging from 100 to 600 mg [25,26].

This review is based on a literature search in PubMed, where approximately 1000 published articles exist for the period of 1950 to 1990 and nearly 6500 for the subsequent 35 years, including over 700 reviews. This indicates the enormous interest in the pharmacological effects and clinical application of ALA. The keywords we used were related to all aspects of preclinical and clinical efficacy and safety data, like lipoic acid or thioctic acid, and pharmacokinetics, pharmacodynamics, safety, toxicity, efficacy, and clinical trials. Additionally, the references of the selected articles were reviewed, and relevant articles were further searched. Only publications in English were considered.

This comprehensive review aims to address the ongoing challenges and concerns related to the use of ALA for treating DPN. We will assess the knowledge gained over the last 70 years while highlighting the existing gaps in ALA’s effectiveness and application in DPN treatment.

## 2. Biosynthesis and Synthesis of ALA

### 2.1. Biosynthesis of ALA

ALA is biosynthesized in mitochondria through sequential steps: mitochondrial fatty acid synthesis type II (FAS II) produces octanoate, which is then transferred to the glycine cleavage H protein as the initial acceptor. Two sulfhydryl groups are subsequently added to the nonpolar octanoic acid side chain to form lipoate, which is finally transferred from the H protein to the E2 subunits of 2-oxoacid dehydrogenase complexes [27]. However, some of these reactions have been poorly characterized, and there are still some open questions.

The first step is the formation of malonyl-CoA, catalyzed in humans by malonyl-CoA synthetase (ACSF3), although alternative sources may exist [28]. Malonyl-CoA:acyl-carrier protein acyltransferase (MCAT) transfers the malonyl group to the acyl-carrier-protein (ACP) [29], followed by a series of enzymatic reactions: condensation to 3-ketoacyl-ACP by 3-oxoacyl-acyl-carrier-protein synthase (OXSM) [30], reduction to 3-hydroxyacyl-ACP by 3-oxoacyl-acyl-carrier-protein reductase [31], dehydration of 3-hydroxyacyl-ACP by 3-hydroxyacyl-thioester dehydratase to 2-trans-enoyl-ACP [32,33], and a final reduction by trans-2-enoyl-CoA reductase which catalyzes the reduction of 2-transenoyl-ACP to acyl-ACP [34]. The product is used for elongation with two further carbons by reaction with another malonyl-ACP at step 3. This cycle repeats three times to yield octanoyl-ACP.

The octanoyl moiety is then transferred to a conserved lysine residue in the glycine cleavage H protein by octanoyl transferase (LIPT2) [35]. This protein-bound octanoate is then sulphurated twice at positions C6 and C8 by lipoic acid (LA) synthetase and produces exclusively the R-enantiomer, which is the biologically active form of LA [36].

The final step involves transfer of the lipoate moiety from the H protein to other mitochondrial proteins requiring lipoylation by lipoyl transferase [35].

ALA cycles between three forms: oxidized (with an intramolecular disulfide bond), intermediate (with substrate bound to one sulfur atom), and reduced (containing two sulfhydryl groups). Dihydrolipoamide dehydrogenase (DLD) regenerates the oxidized form using NAD+ as an electron acceptor, completing the catalytic cycle [37].

Studies in model organisms, particularly yeast, have significantly advanced our understanding of this pathway [38], though some aspects of human ALA biosynthesis remain under investigation. Defects in genes encoding these enzymes can disrupt mitochondrial energy metabolism and cause severe diseases [27].

### 2.2. Synthesis of ALA

While ALA is often produced as a racemate in large-scale manufacturing, the isolation or synthesis of its chiral R-form offers advantages in therapeutic potency for several applications, including its uses as an antioxidant, anti-diabetic agent, and cofactor [8,23,39]. Various approaches have been developed for the synthesis of R-ALA, as described in the comprehensive review of Wang et al. [40].

#### 2.2.1. Chemical Resolution

Industrial production of R-ALA primarily relies on the chemical resolution of racemic ALA using chiral resolving agents. The most common method employs R-(+)-methylbenzylamine (RAMBA) to form diastereomeric salts with ALA, and releasing R-ALA upon subsequent acid hydrolysis [41].

#### 2.2.2. Enzymatic Resolution

Enzyme-catalyzed reactions offer stereoselective alternatives. Lipases from Candida rugosa and Aspergillus oryzae have been used for enantioselective esterification of racemic ALA, though with moderate selectivity toward S-ALA [42]. More efficient approaches utilize enzymatic resolution of precursors like ethyl 8-chloro-6-hydroxy octanoate with Novozym 435, achieving excellent enantiomeric excess (>94% ee) [43].

#### 2.2.3. Chiral Pool Synthesis

Starting from naturally occurring chiral compounds allows for controlled stereochemistry. Examples include syntheses from S-malic acid [44] or D-mannitol [45]. These approaches leverage existing stereochemistry but often require multiple steps.

#### 2.2.4. Chemical Asymmetric Catalysis

Modern asymmetric methods have dramatically improved R-ALA synthesis efficiency. Sharpless asymmetric epoxidation [46], BINOL-Ti-catalyzed allylation [47], and asymmetric hydrogenation using BINAP-Ru catalysts [48] achieve high enantioselectivity. Organocatalytic methods using L-proline have also proven effective [49].

#### 2.2.5. Enzymatic Asymmetric Catalysis

Biocatalytic approaches include Baker’s yeast reduction of β-keto esters [50] and engineered ketoreductases like CpAR2 from Candida parapsilosis, which can reduce ethyl 8-chloro-6-oxooctanoate with >99% ee at industrial scale [41]. Baeyer-Villiger monooxygenases from Pseudomonas species offer another enzymatic route [51].

While chemical resolution remains the primary industrial method for R-ALA production [40], enzymatic approaches—particularly engineered ketoreductases—show promise for more sustainable manufacturing. The highest enantioselectivity (>99% ee) has been achieved using enzymatic reduction with space-time yields exceeding 500 g L^−1^ d^−1^ [41].

R-ALA is the active form synthesized by the body, with absorption enhanced when taken with S-ALA. Breithaupt-Grogler et al. [52] found that volunteers receiving 600 mg of ALA isomers had plasma concentrations of R-ALA nearly 1.5 times higher, although this enantiomer was cleared more rapidly [3]. S-ALA can inhibit the reduction of R-ALA, preventing its binding to reduction enzymes, though this occurs mainly at high S-ALA concentrations. In a racemic mixture, where R-ALA and S-ALA are equal, R-ALA reduction in mitochondria-rich tissues is generally unaffected. This implies that the body’s mechanisms for reducing R-ALA can overcome S-ALA’s inhibitory effects at physiological levels [3].

## 3. Pharmacokinetics

ALA exhibits highly variable pharmacokinetic values that depend on various endogenous and exogenous factors [53,54]. Human studies have shown that ALA is rapidly absorbed, with limited oral bioavailability (~30%) due to its low solubility and short blood half-life, which result from extensive first-pass hepatic metabolism and elevated systemic elimination [8,55,56,57]. There is no evidence that ALA is a substrate of CYP450. The ease with which ALA is metabolized by oxidation, particularly beta-oxidation, is believed to be the leading cause of its unfavorable pharmacokinetic characteristics.

After absorption, ALA enters cells where it is converted into its reduced form, known as DHLA. Depending on the chemical characteristics of the internal environment, either may be present in living organisms. Because both versions can perform biological functions in various settings, they often refer to the pair ALA/DHLA without indicating any distinctions [8,58]. A Tmax peaks within an hour and then declines rapidly [52,57,59,60]. ALA exhibits a high degree of plasma protein binding, primarily to albumin, which affects its distribution and bioavailability. ALA is widely distributed in various tissues, with significant concentrations found in the liver, heart, and skeletal muscle. These tissues are associated with high levels of oxidative metabolism, where ALA functions as a cofactor for mitochondrial enzyme complexes [7]. The ability of ALA to penetrate different tissues and convert to DHLA is crucial for its antioxidant and therapeutic effects. This conversion process, facilitated by redox-related enzymes (such as glutathione reductase, thioredoxin reductase, or dihydrolipoamide dehydrogenase), underscores the importance of ALA in maintaining cellular redox balance. ALA is rapidly eliminated from the body through renal and non-renal routes [3,57,61]. ALA pharmacokinetics remain unchanged by renal clearance, indicating no dose adjustment is needed for patients with renal dysfunction [62].

The significant impact of food on ALA absorption underscores the recommendation to take it on an empty stomach to enhance its bioavailability [62]. The bioavailability and Cmax of ALA are significantly affected and are higher in individuals over 75 than in young adults aged 18 to 45. Age-related pharmacokinetic changes necessitate dosage adjustments in the elderly to prevent potential toxicity [56,63].

## 4. Mechanism of Action

**Figure 1:**
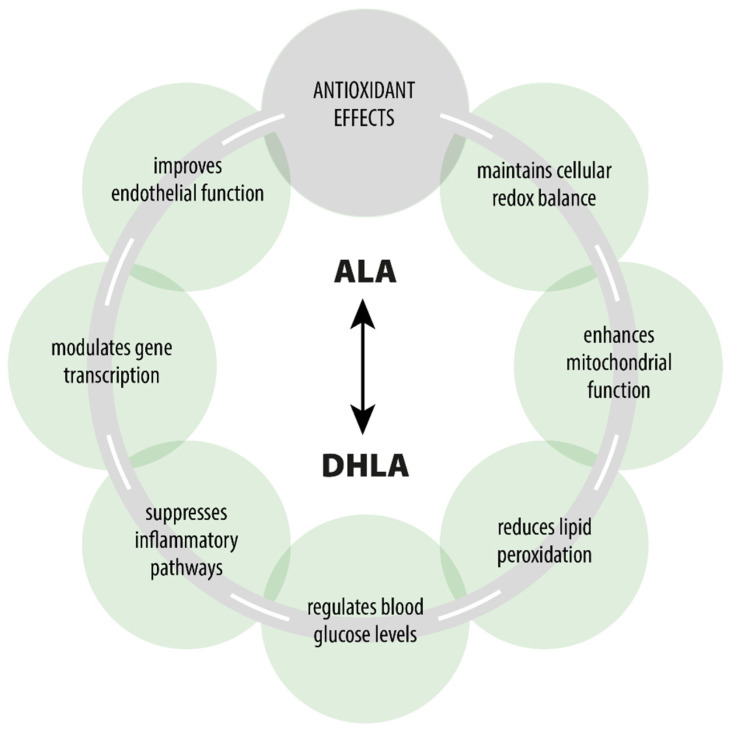
Pharmacological effects of ALA/DHLA.

ALA is a naturally occurring compound that plays a key role in every human cell and exhibits various pharmacodynamic effects beyond its primary role as an antioxidant [7,14,64,65] (Figure 1). ALA participates in the Krebs cycle, fulfills crucial functions in numerous chemical reactions, and serves as a cofactor for certain enzymatic complexes that are essential for energy production within the cell [8].

### 4.1. Antioxidant Properties

**Figure 2:**
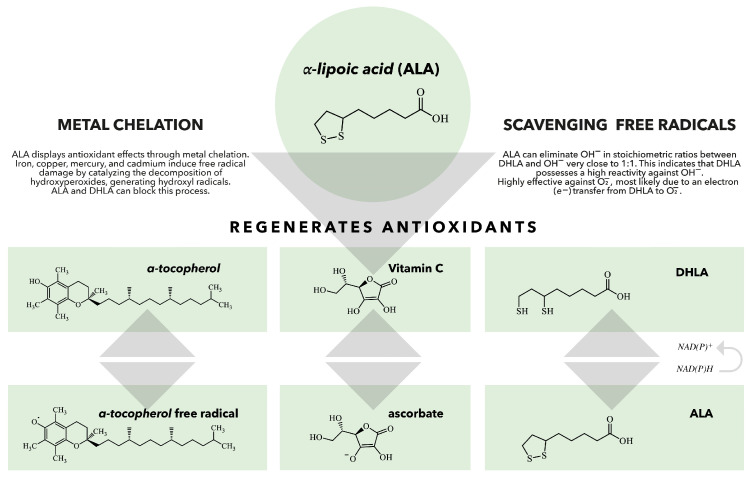
The role of ALA/DHLA in the regeneration of antioxidants. When vitamin E scavenges a peroxyl radical, it produces a vitamin E radical that can be regenerated by various antioxidants, including vitamin C, ubiquinol, and glutathione (GSH). DHLA can reduce all these antioxidants and can also be regenerated by several enzymes, such as lipoamide reductase, GSH reductase, and thioredoxin reductase. This demonstrates that ALA and DHLA play crucial roles in the body’s antioxidant network [75].

There is a vast body of literature on ALA/DHLA antioxidant effects [2,10,66,67,68,69]. ALA scavenges free reactive radicals, which can induce oxidative stress and contribute to various diseases, including diabetes, cardiovascular diseases (CVD), and neurodegenerative disorders [14,15,70,71,72]. ALA serves as both a water-soluble and fat-soluble antioxidant, allowing it to function effectively in both aqueous and lipid environments, such as plasma membranes, cytosol, and mitochondria [68]. It also recycles other antioxidants, such as glutathione (GSH), and vitamins C and E, thereby enhancing their antioxidant capacities and repairing oxidative damage (Figure 2) [14,68,73]. The presence of thiol groups in ALA accounts for its metal-chelating abilities [66]. The interactions of ALA with transition metals are particularly complex, as ALA’s ability to act as an effective scavenger often involves complex synergism with essential trace elements [74]. Moreover, it can increase the levels of GSH within the cells, which chelate and eliminate a wide range of toxins, particularly toxic metals [2,68].

Oxidative stress occurs in experimental diabetic neuropathy (EDN) due to ischemic and autooxidative lipid peroxidation, with resultant neuropathy. Experimental studies in diabetic rats demonstrate that ALA (100–350 mg/kg/day) improves nerve blood flow (NBF), vascular relaxation, and motor nerve conduction velocity while reducing oxidative markers like thiobarbituric acid reactive substances (TBARS) [76]. In lipopolysaccharide (LPS)-induced oxidative stress, ALA (60 mg/kg, IV) treatment alleviated neurophysiological symptoms, normalized TBARS and H_2_O_2_ levels, and improved the GSH/GSSG ratio, indicating enhanced antioxidant defense [77]. Treatment with ALA (20, 50, and 100 mg/kg, IP, five times per week) results in a dose-dependent normalization of GSH in EDN [73]. These findings highlight oxidative stress as a unifying mechanism in neuropathy and support antioxidant interventions, particularly ALA, as a therapeutic strategy.

### 4.2. Regulation of Cellular Redox Status

ALA regulates cellular redox status by modulating the ratio of reduced to oxidized glutathione (GSH/GS-SG) levels, which is critical for cellular defense against oxidative stress [68,73,78]. ALA also enhances the activity of antioxidant enzymes such as glutathione peroxidase and superoxide dismutase. This regulation protects cells from oxidative damage and promotes overall redox homeostasis [3,14]. It is also involved in regulating nuclear factor erythroid 2-related factor 2 (Nrf2), a transcription factor that controls the expression of several antioxidant genes [79]. A systematic review and meta-analysis, including 15 studies, performed by Zonooz et al. [80], suggested that ALA supplementation may improve lipid peroxidation. By inhibiting lipid peroxidation, ALA preserves membrane integrity and protects cells from oxidative injury, which is especially important in DPN, where oxidative damage is a significant pathological contributor [3,14].

### 4.3. Mitochondrial Function and Energy Production

ALA has been shown to protect mitochondrial function. ALA acts as a cofactor in the mitochondrial enzyme complexes involved in the Krebs cycle (e.g., pyruvate dehydrogenase and alpha-ketoglutarate dehydrogenase). It facilitates the conversion of pyruvate to acetyl-CoA, thereby supporting cellular energy metabolism [3,7,14,81]. Supporting mitochondrial function and reducing mitochondrial oxidative stress enhances cellular energy production and prevents energy deficits in peripheral nerves [7,82].

### 4.4. Insulin Sensitivity and Glucose Metabolism

ALA enhances glucose uptake into cells by activating the insulin receptor signaling pathway and improving insulin sensitivity [83,84]. This action reduces insulin resistance and helps regulate blood glucose levels, potentially mitigating hyperglycemia that contributes to nerve damage in DPN [39,69,85].

### 4.5. Effects on Gene Transcription

ALA induces apoptosis in some cancer cells while protecting against oxidative stress-induced apoptosis. It enhances DNA fragmentation and decreases cell viability. Additionally, ALA enhances nuclear levels of apoptosis-inducing factor and activates the caspase-dependent pathway, leading to increased cleavage of cytochrome C and PARP-1. It also activates the caspase-independent pathway by overexpressing poly(ADP-ribose) polymerase. Hence, intracellular Ca2+ mediates both mechanisms of ALA’s apoptotic effects [86].

## 5. Pharmacodynamics

Multiple preclinical studies show agents can prevent or enhance diabetic neuropathy by targeting underlying pathophysiology [87]. ALA has been shown to improve neuropathy symptoms by modulating endoneural blood flow, thereby improving vascular dysfunction, reducing oxidative stress, and counteracting the harmful effects of lipid peroxidation [1,88]. Clinical trials have been carried out on the effects of ALA on DPN, and its use for this condition is approved in many countries [22,89,90,91,92,93,94,95,96].

### 5.1. Neuroprotective Effects

ALA has been demonstrated to have neuroprotective effects through multiple mechanisms. It protects neurons from hyperglycemia-induced damage, reduces advanced glycation end-products (AGEs), and mitigates mitochondrial dysfunction, significantly contributing to DPN [97]. In streptozotocin (STZ)-induced diabetic rats, ALA (100 mg/kg orally) demonstrated superior peripheral nerve protection compared to insulin, improving nerve conduction velocity (NCV) and promoting regeneration, even under persistent hyperglycemia [98]. Additionally, ALA has been shown to upregulate neurotrophins, such as nerve growth factor (NGF) and brain-derived neurotrophic factor (BDNF). These neurotrophins play a crucial role in the survival, repair, and regeneration of peripheral nerves, enhancing neuronal health and functional recovery [99,100,101]. Stevens et al. [102] reported selective effects of ALA (i.p. 100 mg/kg/day for 6 weeks) on STZ-injected diabetic rats. ALA improved digital sensory nerve conduction velocity (NCV) but not sciatic-tibial, corrected endoneurial nutritive but not composite NBF, increased mitochondrial oxidative state without fixing nerve energy depletion, and enhanced polyol pathway intermediate accumulation without depleting myoinositol or taurine. Thus, interactions between nerve biochemistry and perfusion defects from STZ-induced diabetes, corrected by ALA, have differential impacts on nerve fiber populations. Cameron & Cotter [103] studied ALA’s potential for treating diabetic neuropathy by administering it orally at a dose of 100 mg/kg to STZ-induced diabetic rats. The results showed that ALA improved nerve blood flow (NBF) and conduction velocity, which are key indicators of peripheral nerve function. Gorąca & Asłanowicz-Antkowiak [104] found that ALA (60 mg/kg, IV) increased brain-SH groups in LPS-treated rats, demonstrating ALA’s potential to limit nerve tissue damage from oxidative stress. Wang et al. [105] confirm ALA’s neuroprotective effects in a chronic constriction injury model, suggesting its use in chronic neuropathic pain management and supporting its clinical application. However, it showed be noted that in animal models, there is a vast diversity of markers, both in serum (lipid profile, glucose, redox, etc.) as well as in tissues (redox activity—SOD, catalase, glutathione peroxidase—GPx, MDA, carbonylated proteins, AGEs, 4-Hydroxynonenal—4-HNE, among others—inflammatory—TNF-α, IL-1β, IL-6, etc.) or gene expression markers (proteins involved in pro or anti-inflammatory activity, glucose, and lipid metabolism, etc.), in addition to mitochondrial redox markers (8-OHdG, mitochondrial membrane potential, NADPH activity) that have been studied. However, and probably due to this diversity and also the specific characteristics of each tissue analyzed, biomarkers do not always show sensitivity to the ALA action [68,106,107,108,109,110,111].

Clinical studies have indicated that ALA can improve nerve conduction velocity (NCV), restore electrophysiological function in damaged peripheral nerves, and alleviate neuropathic symptoms such as pain, numbness, and burning sensations. This effect is achieved through its antioxidative and anti-inflammatory actions. ALA helps maintain the structural integrity of the myelin sheath, which is essential for efficient nerve conduction [87,112]. It has been explored in treating neuropathy and neurodegenerative diseases [75,113].

### 5.2. Anti-Inflammatory Effects

ALA lowers pro-inflammatory cytokines, such as TNF-α, IL-6, and IL-1β, which are elevated in diabetes, and inhibits the NF-κB pathway [114,115,116,117,118]. By suppressing these inflammatory pathways, ALA alleviates inflammation-induced nerve damage, promotes healing, and prevents further progression of neuropathy [14,64]. Zhang et al. [115] found that ALA (30–120 mg/kg for one week, IP) alleviates neuropathic pain in STZ-induced diabetic rats by downregulating TRPV1 receptors via NF-κB pathways, reducing neuronal excitability. TRPV1 modulation is crucial in diabetic neuropathic pain, involving ROS and cytokine pathways [119,120].

Further studies highlight ALA’s role in neuropathic pain reduction via BDNF-TrkB-ERK signaling [101] and sciatic nerve repair in compression injury models [121].

### 5.3. Effects on Microcirculation

ALA enhances blood flow and oxygen delivery to peripheral nerves by improving endothelial function and reducing vascular dysfunction, which is often compromised in diabetes. It increases nitric oxide (NO) bioavailability by mitigating oxidative stress and preventing NO degradation by ROS. This vasodilatory effect helps restore proper blood flow and oxygen delivery to peripheral nerves, which is essential for nerve repair and function. Additionally, ALA has been shown to decrease vascular endothelial damage by lowering levels of AGEs and inflammatory cytokines, both of which contribute to vascular stiffness and impaired perfusion in diabetes [82,97,122].

In clinical practice, ALA has demonstrated beneficial effects on cardiovascular health, reproductive health, cognitive function, aging processes, detoxification, inflammation reduction, obesity, cancer prevention, and neuroprotection [1,12,122,123,124,125].

## 6. Drug Interactions

ALA is a powerful antioxidant that has been studied for various health benefits; however, it may also interact with certain medications (Table 1). ALA possesses functional pleiotropism across various signal transduction pathways. Evidence is limited that ALA may inhibit specific cytochrome P450 enzymes, potentially altering the metabolism of drugs that are substrates for these enzymes. The inhibition mechanism can relate to the interaction of ALA with sulfhydryl groups due to its dithiolane structure [126]. Makhova et al. [127] demonstrated no significant effect of ALA on individual stages and processes of catalysis of cytochrome P450 3A4, the primary drug-metabolizing enzyme. Phua et al. [128] explored the interaction between ALA (50 mg/kg IV) and valproate (50 mg/kg IV) in rats, finding that ALA increased the AUC 0–6 h for valproate. ALA reduced the in vitro formation of valproate-CoA in a concentration-dependent manner, with no significant distribution-level interactions noted in preliminary protein binding studies. Metabolic stability studies indicated valproate’s metabolism is not primarily eliminated through CYP450, suggesting that the in vivo interaction likely results from inhibition of β-oxidation or glucuronidation. In vitro tests confirmed that ALA significantly inhibited β-oxidation, reducing the metabolic clearance of valproate. Studies on Saccharomyces cerevisiae yeast cells (line D 7) revealed that ALA within the 1 nM–10 μM concentration range does not affect cells’ cytochrome P450 content [129]. However, higher concentrations of ALA (1 mM) proved cytotoxic and destroyed this enzyme [68]. In summary, there is insufficient detailed information regarding ALA’s impact on the metabolism of exogenous substances. It is essential to remember that this process is multifactorial since ALA is present in the diet (as the biologically active form R-ALA), dietary supplements, and drugs. The impact of ALA on metabolism becomes even more complex, given that ALA is an essential endogenous substance for all living cells and functions as a cofactor in various enzyme systems [1]. It is crucial to recognize that ALA may impact liver function in a dose- and concentration-dependent manner, which may also be relevant for potential drug interactions.

ALA may influence GIT motility, potentially affecting the absorption of drugs that require specific GIT conditions for optimal absorption. This is especially true for diabetic patients who experience altered gastric emptying and gastric motility. Delayed gastric emptying, however, does not substantially affect the rate and extent of absorption of both ALA enantiomers [148]. Nevertheless, clinical data on this particular interaction is limited.

## 7. Clinical Implications of ALA

ALA has been used in clinical practice for 70 years to alleviate symptoms associated with various diseases, including neurodegenerative disorders, diabetes, cancer, age-related cardiovascular issues, neuromuscular conditions, weight gain caused by antipsychotic medications, and metabolic obesity [1,8,15].

### 7.1. Effects of ALA on Oxidative Stress

The multifunctional antioxidant properties of ALA highlight its therapeutic potential in oxidative stress-related conditions, particularly DPN [14,64]. Hyperglycemia triggers oxidative stress in mitochondria, which significantly contributes to the development of diabetic microvascular complications [7]. Antioxidants, such as ALA, effectively treat DPN by mitigating oxidative stress [71,122,149,150]. Specifically, ALA enhances GSH, a natural antioxidant involved in antioxidant protection, nutrient metabolism, and the regulation of cellular processes. The RCTs presented in Table 2 clearly demonstrate the effects of ALA on oxidative stress.

The pathogenesis of DPN is influenced by metabolic, vascular, and genetic factors, with oxidative stress playing a significant role in nerve injury [153,154]. DPN is a debilitating condition that adversely affects physical abilities and quality of life [87]. ALA may help correct several mechanisms involved in DPN, including sorbitol accumulation, microvascular damage, oxidative stress, and lipid peroxidation [16]. ALA’s antioxidant properties enhance blood flow, glucose uptake, and energy metabolism [155]. It increases NO production, thereby enhancing endoneural blood flow and potentially alleviating DPN symptoms by protecting endothelial cells and preserving vascular function [82,94,112,156,157]. Additionally, ALA reduces AGEs, which contribute to oxidative stress and neurodegeneration [97,100,122]. By protecting neuronal cells and improving vascular function, ALA helps promote nerve health and function in diabetic animal models [76,158,159].

### 7.2. ALA in the Treatment of DPN

DPN arises from various mechanisms depending on the type of diabetes. It is increasingly common and progresses faster among individuals with Type 1 Diabetes Mellitus (T1DM), with nearly 100% developing DPN after 15 years, compared to approximately 30% in Type 2 Diabetes Mellitus (T2DM) after 25 years [160]. Diabetic neuropathy can affect either the peripheral nervous system, leading to painful diabetic neuropathy [161], or the autonomic nervous system, resulting in diabetic autonomic neuropathy. Autonomic neuropathy can cause life-threatening issues, such as sudden cardiac death [23,110,162]. Moreover, clinical studies have revealed that sex is a significant risk factor for DPN in humans. Research suggests that being male may correlate with a greater risk of developing severe diabetic neuropathy at a younger age. Conversely, men tend to show a lower likelihood of developing neuropathic pain symptoms compared to diabetic women, which reinforces the anti-allodynic effects of testosterone and its derivatives [163].

DPN is characterized by axonal dysfunction and degeneration. It primarily affects sensory nerves, leading to progressive symptoms such as sensory loss, pain, and autonomic dysfunction [96,164]. The pathogenesis of DPN is intricate and remains not completely understood. Several studies have proposed underlying mechanisms, including metabolic, neurovascular, and autoimmune pathways [165,166]. The most widely accepted theory is that oxidative stress is induced in mitochondria by hyperglycemia, which, in turn, causes damage to endothelial and neuronal cells, thereby compromising the oxygen and nutrient supply to the nerves [167]. Macrophages infiltrating nerve cells release cytokines that worsen the damage to nerve fibers [20,22,168]. Other factors contributing to peripheral nerve damage include AGEs and microvascular impairment [20]. These conditions disrupt the expression of neurotrophic factors, leading to oxidative stress and inflammation [150,169,170,171]. Considering all these aspects, antioxidants appear to be a pathogenesis-oriented approach to combat the progression and symptoms of DPN [172,173].

Currently, there is no cure for DPN. Managing DPN remains challenging due to its complex and poorly understood pathophysiology, varied clinical manifestations, and underdeveloped staging [23,174]. Management focuses on slowing progression, relieving pain, and addressing complications [166,175]. Improving glycemic control and making lifestyle changes, including dietary changes and moderate physical activity, are recommended to delay the progression of DPN [18,23,96,164,169,176]. Pain relief options include gabapentinoids (gabapentin, pregabalin), SNRIs (duloxetine, desvenlafaxine), TCAs (amitriptyline), and sodium channel antagonists (oxcarbazepine, lamotrigine, lacosamide, valproic acid), as per the 2022 guideline update [166,177]. Second-line therapies include lidocaine, capsaicin patches, and tramadol [164]. These medications show comparable effectiveness, so factors such as tolerability, contraindications, and cost should be considered when making a choice [22,178]. Essential care includes diabetic foot management, chronic ulcer care, and assessing cardiovascular risks [179]. However, only some patients experience significant relief, often encountering side effects that limit the effectiveness of the treatment. High-dose monotherapy or combinations are used for insufficient pain relief, but tolerability issues limit the benefits, leading to high discontinuation rates [166,180,181,182]. Given DPN’s prevalence and severity, a clear need exists for improved treatments to alleviate the burden on patients and society [18]. Topical medications, behavioral approaches, and physical modalities can be particularly advantageous when combined with other treatments due to their minimal side effect profiles [183,184]. Several non-pharmacological treatments may be utilized in managing DPN; however, most possess a weak strength of evidence [185].

Neuropathic pain-relieving drugs are only modestly effective and often do not eliminate pain, leaving a significant unmet need [181,186]. While complete pain resolution is rare, even a slight reduction in pain can improve quality of life [185,187]. Treatment guidelines typically suggest adding a new agent instead of switching, particularly if the initial medication provided some benefit [87,188,189,190]. A multicenter observational prospective study conducted by Checchia et al. [191] highlighted the potential benefits of a multimodal strategy that combines pharmaceutical, physical, and ALA therapy for the treatment of sciatic neuropathy in real-world settings.

Current guideline-recommended therapies for DPN focus on symptom relief rather than modifying the disease. Aside from glycemic control, there is insufficient data on therapies that can prevent the progression of DPN. Among the pathogenesis-oriented therapies, ALA, actovegin, benfotiamine, and epalrestat are currently authorized for the treatment of DPN in several countries [71,110,192].

**Figure 3:**
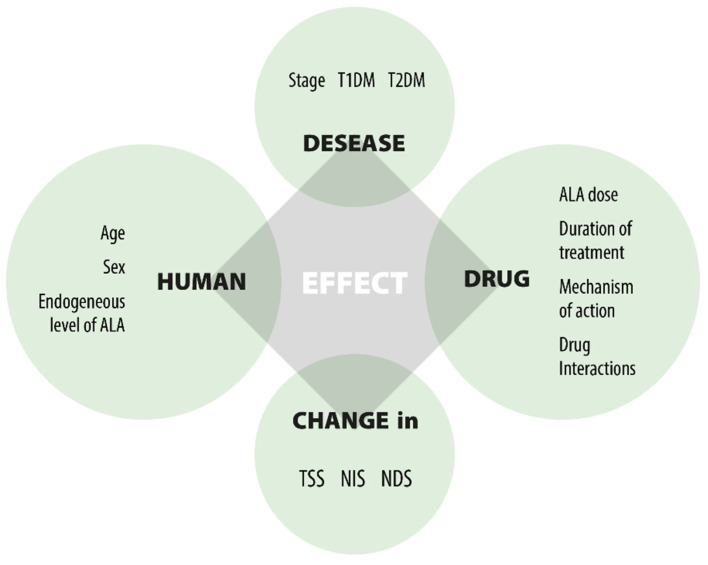
Factors affecting the effect of ALA.

ALA modulates disease and controls symptoms as a therapy focused on pathogenesis. It represents a promising first-line treatment for DPN, preventing early development and progression through both direct and indirect antioxidant effects [8,23,71,87,110] (Figure 3).

Several studies provide comprehensive data on the efficacy and safety of ALA across various dosage regimens and modes of administration. Despite differences in sample size, settings, and study duration, they offer substantial evidence supporting the efficacy of oral ALA at 600 mg/day when administered as a tablet once daily. Outcomes were frequently evaluated using the Total Symptom Score (TSS). The TSS is a summary score that assesses the presence, severity, and duration of lancinating pain, burning pain, prickling (paresthesia), and numbness, with a possible score range from 0 to 14.64 [193]. The data supported the finding that an oral dose of 600 mg/day ALA reduces the primary symptoms of DPN, including pain, paresthesia, and numbness, to a clinically relevant level. Additionally, ALA was well-tolerated throughout the studies, leading to a favorable dose-dependent safety profile (Table 3).

Numerous clinical trials have been carried out to assess the efficacy and safety of ALA in the treatment of DPN. A key confounding factor leading to inconsistent results is the variety of elements that affect clinical responses, as shown in Figure 3. The three main factors are: (1) the unresolved causes of DPN; (2) the multifunctional effects of ALA; and (3) the lack of precise markers to evaluate the drug’s efficacy in DPN treatment. This leads to challenges when comparing outcomes from different clinical studies. Despite differences in study designs and dosing, the significant findings from the trials include: (1) oral ALA at a dose of 600 mg/day effectively improves nerve conduction; (2) the effect is not dose-dependent beyond 600 mg/day; (3) oral ALA at 600 mg/day is equally effective as IV treatment; (4) oral ALA at 600 mg/day is well tolerated; (5) long-term use of oral ALA at 600 mg/day is safe; (6) there is inadequate evidence on the duration of treatment; (7) benefits do not persist after therapy ends; and (8) the clinical efficacy in severe DPN patients remains unverified.

### 7.3. Systematic Reviews and Meta-Analysis

Multiple systematic reviews and meta-analyses have evaluated the efficacy and safety of oral 600 mg/day ALA in treating DPN, showing significant improvements in neuropathic pain and NCV [110].

In a meta-analysis of nine studies, oral ALA treatment, compared to placebo, revealed a reduction in the NIS (muscle weakness, reflex loss, sensation loss), the NIS-LL (motor nerve function and reflexes in the lower limbs), and the TSS [58]. Accordingly, treating DPN with oral ALA is considered a beneficial option, as it alleviates pain symptoms and addresses motor and nerve damage while demonstrating an excellent safety profile [204,205]. Furthermore, individuals with diabetes were found to have lower circulating ALA levels, supporting ALA’s positive impact in the management of DPN [206,207]. In a meta-analysis encompassing 24 randomized controlled trials involving patients with metabolic diseases, ALA was observed to enhance glucose homeostasis (resulting in lower fasting blood glucose, insulin levels, HOMA-IR, and HbA1c) and improve the lipid profile (leading to reductions in triglycerides, total cholesterol, and LDL-C) [208]. Its impact on DPN is thought to be more significant when utilized in conjunction with standard treatments (such as gliclazide, SGLT2 inhibitors, metformin, and GLP-1 analogs) for patients with T2DM who are experiencing neuropathic pain [209]. Abubaker et al. [167] reviewed 8 RCTs involving 1500 patients. The findings were inconsistent regarding the effectiveness of ALA in treating DPN, with some trials observing significant improvements and others failing to present any notable evidence. Nearly 30 systematic reviews and meta-analyses have been published regarding ALA’s beneficial effects in managing diabetes and its complications (Table 4). All studies found ALA to be a safe and tolerable intervention.

In addition to monotherapy, numerous studies indicate that combination therapy may improve clinical symptoms in patients with DPN [95,96,110,146,218,219,220,221]. A meta-analysis of 31 RCTs (n = 2676) evaluated the efficacy and safety of prostaglandin E1 (PGE1) in combination with ALA for treating DPN. The results demonstrate that the combination therapy (ALA/PGE1) is significantly superior to either monotherapy (*p* < 0.00001) [222]. A meta-analysis of 13 RCTs (n = 1148) performed by Jiang et al. [223] reveals that combination therapy with fasudil plus methylcobalamin or ALA is well tolerated and superior to methylcobalamin or ALA monotherapy for improving neuropathic symptoms and NCV in patients with DPN, respectively. Another analysis shows that ALA combined with methylcobalamin improves neuropathic symptoms and NCV more effectively than methylcobalamin alone, without increasing severe adverse events in patients with DPN [224]. The combination of ALA with epalrestat has also been demonstrated to significantly improve clinical outcomes and NCV compared to either treatment alone, with no serious adverse events reported [225,226].

### 7.4. Dosage Regime

#### 7.4.1. Posology in Adults

For adults with sensory disorders caused by DPN, a daily oral dose of 600 mg of ALA is recommended to be taken on an empty stomach (approximately 30 min before the first meal) to enhance absorption [57,85,195,196,227,228,229]. Taking the tablet with food may decrease ALA absorption [6].

If available, initial parenteral therapy with ALA is suggested in cases of severe sensory disturbances [230]. DPN therapy relies on optimal diabetes control. Given the chronic nature of DPN, long-term therapy may be required, along with regular re-evaluation to ensure sustained benefits. Discontinuing ALA after 5 years of treatment led to the recurrence of symptoms after 2 weeks [231].

#### 7.4.2. Posology in the Pediatric Population

There is limited data on the use, efficacy, posology, and safety of ALA in the pediatric population. This limitation can be explained by the fact that ALA is indicated only for adults. However, a few clinical studies have included pediatric patients. In the study by El Amrousy & El-Afify [124], ALA was administered to 40 obese children (ages 10 to 18 years) at a dose of 300 mg twice daily for 3 months. No treatment-related side effects were observed during the treatment period. Hegazy et al. [232] also studied the effects of 300 mg twice daily for four months in 15 asymptomatic T1DM pediatric patients (ages 10–14) to assess potential protective effects on diabetic cardiomyopathy. In the study by Puliappadamb et al. [233], 300 mg/day ALA combined with 5 mg/day flunarizine was administered to 30 adolescents (ages 10–19) for 12 weeks. The ALA/flunarizine combination resulted in a reduction in the frequency and severity of migraines. In a double- blind, parallel-group, placebo-controlled randomized trial, Tromba et al. [234] studied the effect of 800 mg/day ALA for 3 months on cardiovascular risk factors in 32 children aged 8 to 16 with a body mass index greater than the 85th percentile. No adverse events were noted throughout the study period. Scaramuzza et al. [235] evaluated the effects of 400 mg of slow-release ALA, twice daily for 6 months, in 25 T1DM children aged 12 to 19. No severe adverse events were noted during the study period. Huang et al. [236] evaluated the effects of 600 to 1200 mg/day (mean 17 mg/kg/day) on oxidative stress in adolescents (n = 30; mean age 14 ± 2.4) with T1 DM for 3 months. No effects of ALA on oxidative stress were observed, and no adverse events were reported. In the study by Korkina et al. [237], the antioxidant effect of ALA was studied by administering a dose of 400 mg/day for 28 days to 16 children (mean age 11.4 ± 2.1 years) living in the Chernobyl area. ALA significantly lowered urinary radioactivity, normalized liver and kidney functions, and no severe side effects were observed.

## 8. Safety Pharmacology

Clinical and post-marketing surveillance studies have demonstrated the drug’s highly favorable safety profile [1,85,91,201,231,238,239]. To date, neither animal studies [240,241,242] nor human studies have shown serious side effects from administering ALA [3,14,85,194,196,243,244,245]. A meta-analysis of 71 RCTs performed by Fogacci et al. [205] suggests that ALA supplementation of 600 mg once daily is not associated with an increased risk of any treatment-emergent adverse events. In humans, significant toxicity arises at doses of 1200 mg and 1800 mg once daily. Interestingly, ALADIN III study [85] suggested that high doses of ALA are better tolerated when administered in smaller, divided doses.

In a practical cohort of 443 diabetic individuals suffering from chronic painful neuropathy, treatment was administered orally at ALA 600 mg once daily for an average period of five years [231]. Notably, switching from long-term ALA treatment to central analgesic drugs, such as gabapentin, was associated with significantly higher rates of side effects and treatment discontinuation [231]. These findings suggest that DPN treatment is long-term, even during symptom-free intervals, and requires a drug with pathogenetic properties like ALA. Supporting Ruessmann’s [231] data, a retrospective database analysis conducted by Jermendy et al. [204] from 2009 to 2019 revealed a lower occurrence of cardio- and cerebrovascular morbidity, cancer events, and all-cause mortality in DPN patients treated with ALA (n = 23,843) compared to those receiving symptomatic pharmacotherapies (n = 23,843), primarily gabapentin, pregabalin, and duloxetine. Each patient was followed for a minimum of 1 year.

The most frequently reported side effects of oral ALA supplementation are allergic reactions affecting the skin, including rashes, hives, and itching [9]. The use of ALA should be discontinued immediately if any allergic reaction occurs [207]. Abdominal pain, nausea, vomiting, diarrhea, and vertigo have been reported, with one trial indicating that these symptoms are dose-dependent [195]. Additionally, malodorous urine is observed in those taking 1200 mg/day of ALA [246].

Although few adverse effects are noted in animal studies, it has been observed in thiamine-deficient rats that ALA (20 mg/kg IP) caused fatal complications. In contrast, thiamine-sufficient rats showed no adverse effects from ALA supplementation, and the action of ALA in the deficient rats could be prevented by administering thiamine (250 µg) just before ALA administration [144]. The animals in this study were severely thiamine-deficient, exhibiting frank polyneuritis from thiamine deficiency; it may be prudent that any group likely to be severely thiamine-deficient, such as alcoholics, should receive supplemental thiamine if ALA is given.

Additionally, research conducted on primates demonstrated that high doses (90–100 mg/kg IV) resulted in significant necrotic regions in the thigh muscles, as well as in the liver, heart, and kidneys, suggesting that excessive intravenous ALA can cause symptoms identical to those caused by lower doses [247].

### 8.1. Effects on the Cardiovascular System

Several studies have evaluated the effects of ALA on cardiac function. In the longest randomized clinical trial ever conducted [196], oral treatment with 600 mg of ALA once daily for DPN patients showed that the incidence of serious AEs related to cardiovascular disorders, blood pressure (BP), and heart rate did not differ between the ALA and placebo groups. Corrected QT interval (QTc) prolongation > 60 ms occurred significantly more often in the placebo group than in the ALA group (5.0% vs. 1.4%; *p* < 0.05). Dudek et al. (2008) [248] found that ALA (100 mg/kg/day IP for 8 days) did not influence rats’ BP compared to the control group. The same author [249] reported that a single IP dose of ALA 50 mg/kg significantly decreased BP from the 50th minute after drug administration. Consequently, the potential for ALA to reduce BP should be considered, even though it does not cause significant orthostatic hypotension.

In vitro and in vivo studies demonstrated ALA’s protective role in lipotoxic cardiomyopathy [250] and in maintaining cardiovascular function under hypoxic conditions [251,252]. While ALA generally exhibits cardioprotective effects, primarily due to its antioxidant properties, it may provoke adverse cardiovascular effects under certain metabolic conditions [253], particularly in diabetic settings where oxidative stress is poorly regulated.

### 8.2. Effects on the Respiratory System

Years of clinical use have shown that ALA is not expected to negatively impact the respiratory system. Currently, there is no evidence to suggest that ALA itself causes direct adverse effects on lung function or contributes to the development of respiratory diseases. On the contrary, its antioxidant and anti-inflammatory properties seem beneficial for respiratory health, particularly in conditions involving inflammation and oxidative stress [254,255].

### 8.3. Effects on the Central Nervous System

ALA has proven to be safe at the recommended therapeutic dose of 600 mg/day. While ALA has potential therapeutic benefits for improving insulin sensitivity and mitochondrial function, its ability to induce hypoglycemia poses risks to the nervous system, especially under fasting conditions [256,257]. These findings highlight the need for careful monitoring of blood glucose levels when using ALA, particularly in populations at risk of hypoglycemia, as the CNS relies heavily on a constant glucose supply for energy production and normal functioning. ALA can induce refractory convulsions in children in cases of intoxication [258].

### 8.4. Effects on the Liver and Kidneys

A few experimental studies aimed at evaluating the safety of long-term prophylactic treatment with ALA suggest that it may exhibit pro-oxidant properties, cause oxidative stress, disrupt lipid metabolism, and lead to liver steatosis [107,259,260,261,262]. The impact of ALA on methylation pathways suggests a risk of hepatic and renal toxicity, particularly with long-term or high-dose treatments [263]. There is limited clinical experience with patients who have liver impairment. Therefore, when treating individuals with liver issues, ALA should be administered with caution [264].

### 8.5. Reproductive and Developmental Toxicity

The findings from several preclinical studies suggest that ALA plays a protective role in male reproductive health, particularly under oxidative stress conditions and exposure to environmental toxins [265,266,267,268,269]. ALA positively influences embryo development, oocyte maturation, and reproductive outcomes [125,270,271,272,273,274]. Regular ALA administration normalizes menstrual blood flow and reduces pelvic pain in patients with endometriosis. ALA could offer benefits for infertility as a novel agent, and further clinical research is recommended [9,275,276,277]. According to current evidence, using ALA supplements during pregnancy is safe [245,278,279,280]. Di Tucci et al. (2018) [281] performed a comprehensive literature search and concluded that ALA can safely treat neuropathic pain and serve as dietary support during pregnancy. Nonetheless, additional information is necessary to comprehensively grasp the function of ALA supplementation during this time. There appear to be no published reports on ALA levels in human milk, and the excretion of ALA in human milk remains unknown.

### 8.6. Insulin Autoimmune Syndrome

ALA can induce insulin autoimmune syndrome (IAS; also known as Hirata’s disease), characterized by hypoglycemia, high concentrations of immunoreactive insulin, and high titers of antibodies to endogenous insulin, even without prior exposure to exogenously administered insulin. ALA activates the insulin receptor by binding to it extracellularly and can also traverse the cell membrane to activate AMPK, which enhances GLUT4 expression and glucose uptake. This leads to increased glycolysis and initiates the Krebs cycle via interaction with pyruvate dehydrogenase. Additionally, ALA may alter insulin by cleaving disulfide bonds, which exposes fragments to the immune system and results in the production of insulin autoantibodies (IAA), contributing to IAS [65]. IAA is a type of IgG antibody with low affinity and high capacity, causing postprandial and nocturnal hypoglycemia in IAS patients. The IAA titer declines gradually, taking between 2 and 36 months after stopping ALA due to the long half-life (3–4 weeks) of IAA [282].

IAS manifests as neurological symptoms, including tremors, palpitations, anxiety, sweating, hunger, and numbness or tingling. The pathogenesis of ALA-induced IAS remains not fully understood. IAS is classified into two types: type 1, caused by antibodies against insulin [283], and type 2, caused by antibodies against the insulin receptor [282]. An individual’s ethnic and genetic background significantly influences their predisposition to IAS [284,285,286]. Individuals with HLA-DRB1*04:03 and HLA-DRB1*04:06 who consume ALA might have a higher risk of developing IAS [284,286,287]. When conducting a differential diagnosis of spontaneous hypoglycemia in individuals taking ALA, IAS should be considered [65,284,288,289]. Since IAS is a type VII hypersensitivity autoimmune disease, it is assumed that the consumed dose does not significantly contribute to the development of IAS. The presence of other autoimmune diseases may predispose to the development of IAS. Drug-induced cases have been reported, linked to carbimazole, methimazole, clopidogrel, propylthiouracil, isoniazid, hydralazine, imipenem, and ALA [283,286,290,291]. However, IAS is not associated with consuming naturally occurring ALA-containing foods [288].

ALA’s disulfide bonds and sulfhydryl (-SH) groups may contribute to its potential to induce autoimmunity through mechanisms such as hapten formation or alterations in protein structure and antigenicity. Li et al. (2021) [292] investigated the clinical characteristics of ALA-induced IAS in 37 patients (23 from Asia, 13 from Europe, and one from South America). The median age of these patients was 61 years (range 32–82), and the median BMI was 26.3 kg/m^2^ (range 17.8–36.9). The median dose was 600 mg (range 200–600), with a median course of treatment lasting 20 days (range 2–120) and administered through PO or IV routes. Six patients (16.2%) also had autoimmune diseases, including rheumatoid arthritis, anaphylactoid purpura, autoimmune thyroiditis, and glomerulonephritis.

IAS can develop within 1 week to 2 months during ALA use and may also appear a few hours to 2 weeks after discontinuing the medication [282]. IAS is a self-limiting condition with a favorable prognosis. Symptoms of hypoglycemia typically resolve within 3 to 8 months following the cessation of ALA. Steroid treatment and the discontinuation of ALA will, over time, cause IAS to disappear [293]. Refractory cases may require immunomodulators such as rituximab or azathioprine [294]. There is likely an underestimation of IAS occurrences due to a lack of awareness about the disease, resulting in underdiagnosis and underreporting. Due to the limited data available, it is impossible to accurately quantify the risk of developing IAS after consuming ALA. The safe dose of ALA that would not lead to IAS may vary among individuals and cannot be determined based on the current evidence [288].

### 8.7. Overdosage

The range of toxicity from ALA ingestion is varied and challenging to determine based on existing literature. ALA is generally considered very safe, with intoxication being quite rare [295]. The initial clinical signs of intoxication include psychomotor agitation or impaired consciousness, often accompanied by generalized convulsions and the onset of lactic acidosis [296]. Seizures that progress to status epilepticus have been reported in pediatric patients [258,297,298,299]. Polat et al. [299] presented a case of ALA intoxication in a 16-year-old girl who took a total dose of 1800 mg ALA. Furthermore, hypoglycemia, shock, rhabdomyolysis, hemolysis, disseminated intravascular coagulation, bone marrow depression, thrombocytopenia, and multiple organ failure have been recognized as consequences of high-dose ALA intoxication [298,299,300,301]. The initiation of general poisoning therapy (such as induced vomiting, gastric lavage, and activated charcoal administration), along with prompt hospitalization, is advised if there is even the slightest suspicion of ALA intoxication (for instance, more than 6 g in adults or over 50 mg/kg in children). Treatment for lactic acidosis, generalized seizures, and other potentially life-threatening effects should be symptomatic and follow current intensive care unit guidelines. To date, there is no evidence that hemodialysis, hemoperfusion, or filtration methods are effective in facilitating the removal of ALA.

## 9. Market Share of ALA

Alpha-lipoic acid is a common ingredient in various multivitamin products, nutritional supplements, cosmetics, and prescription drugs. ALA is available for topical, oral, and intravenous administration. The pharmaceutical segment dominates the global ALA market [302,303,304,305]. As a prescription drug, ALA is offered only at a dose of 600 mg for oral or intravenous administration. As mentioned earlier, a dose of 600 mg once daily has been established as effective and well-tolerated in numerous randomized clinical trials for treating DPN. In contrast, as a nutritional supplement, ALA is found as a mono-ingredient product at various doses, ranging from 100 to 600 mg. ALA is also available in combination products, typically containing B vitamins, microelements, and various plant extracts. In these combined nutritional supplements, ALA is present in the range of 7 to 600 mg. This significant variation in the amount of ALA in nutritional supplements serves as the basis for discussion regarding the awareness of patients and health professionals. Six drug formulations (four as oral tablets or capsules and two as solutions for injection) and ten dietary supplements are available on the Bulgarian market. All four drug formulations contain only 30 tablets or capsules of 600 mg ALA, while the nutritional formulations contain between 30 and 240 tablets or capsules. On the Bulgarian market, only two nutritional supplements contain the active, naturally occurring enantiomer, e.g., R-ALA. All the others contain the racemic ALA. In contrast, R-ALA is the most common product in the global ALA market [302,305].

The global ALA market was valued at 784.5 million dollars in 2020, 910.3 million dollars in 2023, and 958.3 million dollars in 2024. The global ALA market is estimated to be 1146.5 million dollars in 2025 and is expected to reach 1854.3 million dollars by 2032, demonstrating a CAGR of 7.1% from 2025 to 2032 [303,305].

North America dominates the ALA market due to increased public awareness of its health benefits, a strong presence of suppliers and manufacturers in the region, and a well-established market for supplements [304,305]. North America will account for 34% of the market share in 2025. Europe is the second-largest ALA market in the world, with Germany being a leading country; however, the Asia-Pacific market is expected to experience the highest growth over the next decade [270,271]. In 2020 and 2021, this market accounted for 29.9% and 33.4% of ALA market share [305]. China is the second-largest consumer of cosmetics in the world, after the US, and has reported an increased demand for ALA to manufacture anti-aging, sun protection, and other products [302,303]. The US ALA market is expected to grow at a CAGR of 5.8%, Europe at a CAGR of 5.5%, and Asia-Pacific at a CAGR of 6.9% from 2024 to 2030. This makes the Asia-Pacific market the fastest-growing of the three [303,305].

## 10. Discussion

Alpha-lipoic acid has been extensively studied for over 70 years, yet significant gaps remain in our understanding of its optimal clinical application for DPN. This comprehensive review reveals several critical considerations that merit further discussion.

The pleiotropic nature of ALA presents both opportunities and challenges in therapeutic applications [8,9,64]. As an endogenous substance with universal antioxidant properties, ALA’s mechanism of action in DPN is multifaceted, involving antioxidant effects [14,15,68], regulation of cellular redox status [68,73,78], improvement of mitochondrial function [3,7,81,82], enhancement of insulin sensitivity [65,83,84], and modulation of inflammatory pathways [114,115,116,117,118]. This complexity makes it difficult to isolate the precise mechanisms responsible for its clinical benefits in DPN. Furthermore, the interaction between ALA and the pathophysiology of DPN, which itself is incompletely understood [165,166], creates additional layers of complexity.

Clinical evidence supports 600 mg/day oral ALA as an effective and well-tolerated treatment for DPN, with efficacy comparable to intravenous administration [94,110,195,211]. However, the optimal treatment duration remains uncertain. While some studies suggest benefits within 3–5 weeks [229], others indicate that long-term therapy (≥6 months) may be necessary for sustained improvements [85,196]. The NATHAN 1 trial’s four-year data demonstrated that ALA had a favorable impact on neuropathic impairments but not on neurophysiological markers [196], suggesting that different aspects of DPN may respond differently to ALA therapy. Additionally, evidence suggests that discontinuation of ALA leads to symptom recurrence [231], indicating that ongoing treatment may be necessary.

The variability in ALA’s pharmacokinetics presents practical challenges for clinical application [53,54]. Factors such as food intake [57,62], age [56,63], genetic variations [284,286,287], and concurrent medications [126,127,128] can significantly affect ALA’s bioavailability and efficacy. The high first-pass metabolism and short half-life of ALA necessitate careful consideration of dosing regimens [52,57,59,60]. Furthermore, the different forms of ALA available commercially (racemic mixture versus R-enantiomer) [25,26] may have different pharmacokinetic profiles and therapeutic efficacies, yet most clinical studies have used the racemic form [302,305].

Safety considerations reveal that while ALA is generally well-tolerated at 600 mg/day [205,216], higher doses may increase the risk of adverse effects [195]. The potential for rare but serious reactions such as IAS [65,282,283,284,285,286], particularly in individuals with specific HLA genotypes [284,286,287], warrants caution. The risk of hypoglycemia, especially when ALA is combined with antidiabetic medications [130,131,132], necessitates careful monitoring. Moreover, the potential pro-oxidant effects of ALA at high doses highlight the importance of appropriate dosing [107,259,260,261,262].

A significant limitation in the current evidence base is the heterogeneity in study designs, patient populations, outcome measures, and treatment protocols [22,193,213]. Many trials have been relatively short-term, potentially underestimating both benefits and risks of long-term ALA therapy [85,194,195,196]. The lack of standardized assessment tools for DPN severity and progression complicates the interpretation of treatment effects across studies [23,174]. Additionally, most studies have focused on symptomatic improvements rather than disease modification or prevention [22,175].

The expanding market for ALA presents challenges related to product quality, standardization, and regulatory oversight [302,303,304,305]. The availability of ALA as both a pharmaceutical and a dietary supplement creates potential confusion among healthcare providers and patients regarding appropriate formulations, dosing, and quality standards. The significant variations in ALA content in nutritional supplements (7–600 mg) compared to the established therapeutic dose (600 mg) may lead to suboptimal treatment outcomes.

Future research should focus on identifying biomarkers that predict response to ALA therapy [87,110], optimizing dosing regimens based on pharmacokinetic/pharmacodynamic modeling [53,57], and developing extended-release formulations to overcome the limitations of ALA’s short half-life [59,60]. Longitudinal studies investigating the preventive potential of ALA in early-stage DPN [71,87] and studies comparing R-ALA to racemic ALA in clinical settings are warranted [25,56]. Genetic studies could help identify individuals at risk for adverse effects such as IAS [284,286,287]. Additionally, combination therapies that leverage synergistic effects between ALA and other pathogenetically oriented treatments deserve further investigation [95,96,146,218,219,220,221,222,223,224,225,226].

## 11. Conclusions

Scientific literature offers extensive information on ALA’s pharmacodynamics, pharmacokinetics, efficacy, and safety. ALA represents a valuable therapeutic option for managing DPN, offering benefits through multiple mechanisms that address the pathophysiological processes underlying diabetic neuropathy. The evidence supports oral ALA at 600 mg/day as an effective and well-tolerated treatment that improves neuropathic symptoms. The favorable safety profile of ALA at this dose makes it an attractive option, particularly for long-term management. However, several challenges remain, including optimizing treatment duration, addressing pharmacokinetic variability, managing potential drug interactions, and minimizing rare adverse effects. The heterogeneity in available ALA products and varying regulatory standards across regions complicates clinical implementation. Despite these challenges, ALA holds promise as a pathogenesis-oriented therapy for DPN, offering advantages over purely symptomatic treatments. As our understanding of both ALA and DPN continues to evolve, improved clinical protocols and more targeted approaches may enhance the effectiveness of ALA therapy. Future research should focus on personalized treatment strategies, biomarker development, and combination therapies to maximize the therapeutic potential of this endogenous antioxidant that has withstood 70 years of scientific scrutiny.

## 12. Limitations

A key limitation of this review is the challenge of covering all areas related to the biological effects and medical implications of ALA. We have endeavored to cite many distinguished reviews; however, several aspects of synthesis, natural sources of ALA, and its applications in various medical and cosmetic fields remain unaddressed in this review, though they are covered by the cited reviews.

## Figures and Tables

**Table 1:** Drug Interactions of ALA.

Drugs [References]	Effects of ALA
Insulin and Oral Antidiabetic Drugs [65,130,131,132]	Enhances insulin sensitivity, amplifies the effects of insulin and other antidiabetic medications (e.g., metformin, sulfonylureas), and raises the risk of hypoglycemia.
Antioxidants [68,133]	They may have synergistic effects, but antagonistic interactions might also impact the pharmacodynamics of these supplements.
Metal-binding Drugs [66,68,133]	May affect the effectiveness of certain metal-based medications or supplements, including some antibiotics and cancer treatments.
Chemotherapy Drugs [123,134,135,136,137,138,139]	May hinder the effectiveness of certain chemotherapy drugs, particularly those that produce free radicals. Its antioxidant properties might diminish the efficacy of chemotherapeutic agents that operate through oxidative stress. Some studies indicated that ALA could enhance the effect of certain chemotherapeutics by reducing oxidative damage to normal cells. ALA has been shown to counteract the adverse effects of anticancer agents, including neuropathy.
Iron/Calcium/Magnesium Supplements [68,134,140,141]	This may decrease their absorption and effectiveness. This interaction is relevant only to products administered orally. Supplementation with ALA did not show a statistically significant impact on iron-related measures. Analysis of subgroups indicated a notable increase in ALA’s effect on hemoglobin among patients with hematological conditions and in studies that lasted longer than eight weeks.
Thyroid Medications [134,142,143]	It may impact thyroid hormone levels and affect the efficacy of thyroid medications in some individuals.
Alcohol [68,144,145,146]	Chronic alcohol consumption may decrease the effectiveness of ALA, as it can impair the absorption and utilization of antioxidants. In addition, alcohol can lower the amount of thiamine (vit. B1) in the body. Thiamine is crucial for nerve function and energy metabolism in the brain. Thiamine deficiency, if induced or exacerbated by high-dose ALA, can lead to conditions such as Wernicke-Korsakoff syndrome, which manifests in CNS-related symptoms like confusion, ataxia (lack of coordination), and memory problems. However, this is more of a concern for individuals already thiamine-deficient than for the general population.
Biotin [14,64,147]	The chemical structure of biotin is similar to that of ALA, and there is some evidence that high concentrations of lipoic acid can compete with biotin for transport across cell membranes.

**Table 2:** RCTs exploring the effects of ALA on oxidative parameters.

Participants/Patients Treated with PO 600 mg ALA	ALA Dose/Duration of the Study	Main Outcomes	Safety
A randomized, placebo-controlled study [151]			
29 T2DM (age > 18)	300 mg/day for 8 weeks	The ALA group showed a significant decrease in FBG and PPG levels, IR-HOMA index, and GPx levels. There is a significant difference between FBG and IR at the beginning and end of the study in the ALA-treated and PLA-groups.	ND
A randomized, double-blinded, placebo-controlled study [152]			
30 T2DM (age > 18)	300, 600, 900, 1200 mg/day for 24 weeks	FBG and HbA1c trended to decrease in a dose-dependent manner. An increase of urinary F2α-IsoP was noted in PLA but not ALA-treated groups, suggesting a potential inhibitory effect of ALA on lipid peroxidation in individuals with DM.	Well tolerated; minor side effects (1 patient—anorexia; 2 patients—skin rash)

Abbreviations: ALA, alpha lipoic acid; DM, diabetes mellitus; FBG, fasting blood glucose; GPx, glutathione peroxidase; HbA1c, glycated hemoglobin; IR-HOMA, Homeostatic Model Assessment for Insulin Resistance; ND, no data; F2α-IsoP, PGF2α-Isoprostanes; T2DM, type 2 DM; TGF-β, transforming growth factor beta.

**Table 3:** Summary of the trials investigating the efficacy and safety of oral ALA.

Study [Reference]	Population	Duration	Intervention	Control	Efficacy	Safety	Issues
Randomized controlled trials							
ALADIN II [194] Multi-center, prospective, randomized, double-blind, placebo-controlled clinical trial	299 T1/T2 DM participants with mild to moderate DPN and on NCS (age 18–60; mean 57.8 ± 9.73)	96 weeks (2 years)	ALA of 1200 or 600 mg/day or PLA was IV administered for 5 consecutive days before enrolling the patients in the long-term PO phase. 18 given ALA 1200 PO (G1: 6 × 200 mg), 27 given ALA 600 PO (G2: 3 × 200 mg + 3 × 200 mg PLA)	20 given PLA tablets (G3: 6 × 200 mg PLA)	2-year treatment may exert favorable effect on the peripheral nerve function of DM patients; Neurophysiologic markers: sural NCV statistically significant improvement for ALA1200 and ALA600 vs. PLA. NSD between the three groups were noted for NDS changes from baseline to 24 months (−0.2 ±2.9 points in ALA 1200, −0.19 ± 2.13 points in ALA 600, and −0.6 ± 3.1 points in PLA).	Treatment-emergent AEs and laboratory tests showed no differences between the groups. The global assessment of tolerability was very good and/or good in 100% of the patients in the PLA group, 89% in ALA 600 and 94% in ALA 1200.	Multicenter (32 outpatient centers) nature increases variability of results. Major problems were faced even before completion of the study, including a high rate of drop-outs (n = 52), withdrawal due to concurrent disease (n = 15) or AEs (n = 3), protocol violators (n = 31), and patients with peripheral vascular disease (n = 29). The primary analysis, therefore included 169 patients, who had completed the 24-month trial.
ALADIN III [85] Multicenter, randomized, double-blind, placebo-controlled clinical trial	503 T2DM participants with TSS > 4 and NIS > 2 (age 18–65; mean 56.9 ± 6.23)	24 weeks	ALA-ALA group (n = 165): 600 mg ALA once daily IV for 3 weeks, followed by 600 mg ALA t.i.d PO for 6 months	ALA-PLA group (n = 173): 600 mg ALA once daily IV for 3 weeks, followed by PLA t.i.d PO for 6 months PLA-PLA group (n = 165); PLA once daily IV for 3 weeks, followed by PLA t.i.d PO for 6 months	Could not demonstrate any effect after 19 days; mean TSS change from baseline to day 19: ALA600: −3.7 (−12.6 to 5), PLA: −3 (−12.3 to 8), NSD; TSS after 7 months: NDS between 2 groups; mean NIS change from baseline to day 19: ALA600: −4.34 ± 0.35, PLA: −3.49 ± 0.58, *p* = 0.02	During the oral treatment phase, the rates of AEs were 77/167 (46.1%) in ALA-ALA, 66/174 (37.9%) in ALA-P, and 75/168 (44.6%) in P-PLA, with NSD between the groups.	Multicenter (71 outpatient centers) Nature of the study increases variability of results. The total withdrawal rate was 25% with NSD between the groups.
SYDNEY 2 [195] Multicenter, randomized, double-blind, placebo-controlled clinical trial	181 T1/T2 DM participants and TSS > 7.5 and NIS-LL > 2 (age 18–74; mean 57.5 ± 11)	5 weeks	45 given ALA600 PO, 47 given ALA1200 PO, 46 given ALA 1800 PO	43 given PLA	Efficacy of PO ALA600 on neuropathic pain is comparable with ALA600 IV; PO ALA600 is the most appropriate dose; TSS mean change from baseline to end of study: ALA600 vs. ALA1200 vs. ALA1800 vs. PLA (−4.9 vs. −4.5 vs. −4.7 vs. −2.9, *p* < 0.05 vs. PLA)	TEAEs were 21% in the PLA group, 27% in the ALA600 group (NSD), 43% in the ALA1200 group (*p* < 0.05 vs. PLA), and 54% in the ALA1800 group (*p* < 0.05 vs. PLA).	15 (8%) subjects discontinued during the treatment period because of AEs: 1 in PLA group, 0 in the ALA600, 5 in ALA1200, and 6 in ALA1800.
NATHAN 1 [196] Multicenter, randomized, double-blind, placebo-controlled clinical trial	460 T1/T2 DM participants with mild-to-moderate DPN and TSS < 5 NIS-LL > 2 (age 18–64; mean 53.6 ± 7.95)	216 weeks (4 years)	231 given ALA 600 mg/day PO	225 given PLA	ALA600 PO for 4 years was associated with the improvement of neuropathic impairments but not neurophysiologic markers. TSS and NIS change at 4 years: ALA vs. PLA (NSD)	GAT by investigators and patients showed NSD between the groups. Serious AEs occurred in 38.1% of patients in the ALA group and 28.0% of PLA group.	Two (0.9%) patients on ALA and one (0.7%) on PLA discontinued the study as a result of lack of tolerability (“likely” causal relationship to study medication as judged by the investigator).
India ALA [197] A randomized, open-label, placebo-controlled trial	20 T2DM with DPN (age 40–65)	12 weeks	10 given PO 600 mg/day ALA	10 given PLA	NCV significantly increased in the ALA group. ALA slows the progression of nerve degeneration and improves patient compliance; however, it does not alter glycemic control.	NDA	Open-label design
Egypt ALA [198] A prospective, double-blind, placebo-controlled trial	200 T2DM with DPN	24 weeks	100 given 600 mg ALA b.i.d.	100 given PLA	Improvement in NSS after 6 months treatment (60.9 ± 32.9% for ALA vs. 23.2 ± 14.1% for PLA). NDS and VPT improved after 1 month of treatment. Reduction of pain was not evident by VAS.	No AEs were reported.	Various assessment scales should be considered when interpreting the data.
Korea 2 ALA [199] A double-placebo, randomized, noninferiority trial	100 T2DM with DPN	12 weeks	Active comparator: 52 given PO 600 mg/day ALA + PLA b.i.d	48 given GLA 320 mg/day b.i.d + PLA q.d.	The mean VAS score at baseline was 5.58 ± 1.35, compared to 3.92 ± 2.12 after 12 weeks of treatment. The TSS significantly decreased from 5.15 ± 3.35 at baseline to 3.52 ± 3.39 after 12 weeks of treatment (*p* < 0.001).	Patients tolerated the treatment well, as no new safety concerns or events related to ALA were reported.	73 completed the 12-week treatment period
Pakistan ALA [200] A randomized controlled trial	110 T1/T2 DM with TSS ≥ 4. (age 20–70; mean 46.88 ± 11.26)	24 weeks	55 given PO 600 mg/day ALA	55 in the control group	The mean change in TSS in the treatment group was 2.38 ± 1.99, and in the control group was 0.53 ± 1.32 (*p* < 0.001). The comparison of TSS was significantly reduced for other variables (HbA1c, numbness, burning, and paresthesia) in the post-treatment group than the pretreatment group (*p* < 0.001).	No AEs were reported ALA was well tolerated, and no patient discontinued treatment.	The control group is not strictly defined. PLA treatment is not included.
Uncontrolled studies							
Korea ALA [201] Single-center, open-label clinical trial	61 DM with mild to moderate DPN and abnormal NCS and TSS ≥ 6 (age 18–70; mean 58.7 ± 6.2)	8 weeks	38 given ALA 600 mg PO once daily		The response rates increased during the study, achieving 47.4% at 4 weeks and 71% at 8 weeks; improvement in TSS: responders (n = 27) vs. non-responders (n = 11) (57.3 ± 15.93% vs. 15.44 ± 14.05%, *p* = 0.01)	86.8% (33/38) and 76.3% (29/38) had good or satisfactory efficacy at the end of the study, as rated by the physician and patients, respectively. Global tolerance was rated as good or satisfactory by physicians and all 38 patients.	Among the 23 (37.7%) withdrawals, 17 (27.9%) dropped out due to protocol violation, 5 (8.2%) patients due to AEs, and 1 (1.6%) patient due to consent withdrawal.
Mexico ALA [202] A multicenter randomized withdrawal open-label study	45 T2DM with DPN and TSS > 7 who responded to (Phase 1) (mean age 58.2 ± 10.5)	20 weeks	Phase 1: Initial 4-week high LD of 600 mg ALA t.i.d. to determine responders. Responders who decreased ≥3 TSS points after phase 1 were randomized to receive 600 mg/day of ALA orally for 16 weeks or ALA withdrawal. Phase 2: 16 were given 600 mg/day for 16 weeks, and 17 controls (withdrawal group) were given PLA t.i.d.		Responders During phase 1, the TSS decreased from 8.9 ± 1.8 to 3.46 ± 2.0. During phase 2, TSS improved from 3.7 ± 1.9 points to 2.5 ± 2.5 in the ALA group (*p* < 0.05) and remained unchanged in the ALA withdrawal group.	No TEAEs were observed throughout the study.	No PLA treatment during phase 2 of the study; compared responders vs. nonresponders.
Egypt 2 ALA [203] A prospective, interventional study	90 T2DM with DPN (age 50–60.3; mean 52)	12 weeks	90 given PO 600 mg/day ALA (compared to pre- and post-treatment)		ALA significantly improved NCV, LDL, HDL, HbA1c, and BMI. Failed to prove the effect of ALA on the nerve cross-section area.	NDA	It is unclear whether the improvements are related to ALA or to the improvement in glycemic control.

Abbreviations: AEs, adverse events; ALA, alpha lipoic acid; BMI, body mass index; BPI, brief pain inventory; DM, diabetes mellitus; DN4, douleur neuropathique 4; DPN, diabetic peripheral neuropathy; GAT, global assessment of tolerability; HbA1c, glycated hemoglobin A1c; HDL, high density lipoproteins; LD, loading dose; LDL, low density lipoproteins; IV, intravenous; NCS, nerve conduction studies; NCV, nerve conduction velocity; NDA, no data available; NDS, neuropathy disability score; NIS, neuropathy impairment score; NIS-LL, neuropathy impairment score-lower limbs; NPSI, neuropathic pain symptom inventory; NSD, no significant difference; NSS, neuropathy symptom score; PGI-I, patient global impression-improvement; PLA, placebo; PO, per oral (per orem); SDS, sheehan disability scale; SPNSQ, subjective peripheral neuropathy screen questionnaire; SNCV, sensory nerve conduction velocity; T1DM, diabetes mellitus type 1; T2DM, diabetes mellitus type 2; t.i.d., three times a day (ter in die); TEAEs, treatment emerged AEs; TSS, total symptom score; VAS, visual analogue pain scale; VPT, vibration perception threshold.

**Table 4:** Systematic reviews and meta-analyses establishing DPN treatment’s effectiveness and safety, including ALA (listed by year).

Reference	Focus	Clinical Trials	ALA Efficacy Findings	ALA Safety Findings
Ziegler et al. [91]	A comprehensive systematic review and meta-analysis of antioxidant therapy with ALA in DPN.	4 RCTs (n = 1258)	ALA (600 mg/day PO) significantly improved TSS, reducing neuropathic deficits and symptoms, such as pain and burning sensations.	ALA was generally well-tolerated, with minimal AEs, which were mostly mild GIT symptoms.
Mcllduff & Rutkove [89]	A critical appraisal of IV and PO ALA in treating symptomatic DPN.	5 RCTs (n = 1160)	ALA (600 mg/day PO) for up to 5 weeks) demonstrates beneficial effects for managing DPN.	No significant AEs were reported.
Mijnhout et al. [210]	Systematic review and meta-analysis of RCTs on ALA in DPN.	4 RCTs (n = 653)	A significant and clinically relevant decrease in neuropathic pain when administered for 3 weeks at 600 mg/day (grade A recommendation).	AEs were mild, including minor GIT disturbances, similar to those in PLA groups. No serious AEs were reported.
Han et al. [154]	A systematic review and meta-analysis to evaluate the efficacy and safety of ALA in treating DPN.	15 RCTs (n = 1052)	ALA (300–600 mg/day i.v. for 2–4 weeks) significantly improves NCV and neuropathic symptoms. Nonetheless, the evidence might not be robust due to the poor methodological quality of the studies included in this review.	ALA is a safe option for managing DPN, but it is emphasized that the higher doses result in increased rates of GIT side effects.
Çakici et al. [211]	A systematic review and meta-analysis to evaluate the efficacy and safety of various treatments for DPN.	27 RCTs; 19 different interventions; ALA treatment (6 studies)	ALA, along with other treatments, had significant beneficial effects on managing DPN symptoms. Significant improvements in TSS were observed compared with PLA in five trials. Oral 600 mg/day ALA affected DPN symptoms, identical to those of IV ALA treatment.	ALA was generally well-tolerated. The most common AEs were mild GIT issues, as nausea and abdominal discomfort. However, these AEs were not severe and did not result in treatment discontinuation for most patients.
Dy et al. [193]	Preventing Complications and Treating Symptoms of DPN.	62 RCTs and nonrandomized studies for prevention or treatment of DPN symptoms	5 RCTs: ALA was more effective than PLA in reducing pain, although the studies were short-term (<3 months) and had a low SOE. Inconsistency across the studies and unclear risk of bias.	Specific adverse effects occurring in more than 10% of patients in at least one study arm receiving ALA included nausea (1% to 25%), vomiting (0% to 26%), and vertigo (4% to 11%). Rates were dose-dependent, with the highest rates in the 1800 mg group.
Nguyen & Takemoto [212]	Evaluation of efficacy, safety, and cost of ALA compared to other DPN treatments.	25 RCTs and 3 open-label studies	Although studies on ALA provided lower strength of evidence, given the limitations of other pharmacologic approaches, ALA could be of particular value. Current data provides evidence of ALA’s benefits in DPN treatment at 600 mg/day, IV or PO, for at least 3 weeks.	Minimal side effects compared to other pharmacological treatments for DPN, such as gabapentin and duloxetine. Favorable cost and tolerability of ALA compared to other DPN treatments.
Amato et al. [213]	Follow-up ranged from 3 weeks to 4 years, with 4 RCTs 5 weeks or less in duration	23 RCTs assessing non-pharmacologic intervention therapies for DPN	6 RCTs: ALA was more effective than PLA for the outcome of pain (low SOE)	3 RCTs of ALA reported adverse effects. Rates occurring in more than 10% of participants in at least one study arm included nausea (ranging from 1% to 25%), vomiting (ranging from 0% to 26%), and vertigo (ranging from 4% to 11%). Rates were dose-dependent, with the highest rates in the 1800 mg group.
Fogacci et al. [205]	A systematic review and meta-analysis of the side effects of ALA from the available RCTs	71 clinical studies, comprising 155 treatment arms, which included 2558 subjects treated with ALA and 2294 assigned to PLA	Not evaluated	ALA was safe and not associated with an increased risk of any TEAE.
Jibril et al. [214]	A Cochrane systematic review and meta-analysis of ALA effects on cardiometabolic risk factors in patients with T2DM	16 RCTs (n = 1035)	Although statistically significant effects of ALA supplementation on cardiometabolic risk factors were found, these effects were smaller than MCID thresholds for all primary outcomes.	Minor adverse events, including anorexia, diarrhea, heartburn, and other GIT problems.
Hsieh et al. [175]	A systematic review and meta-analysis to evaluate the effects of oral ALA on DPN	10 RCTs (n = 1242)	ALA (600 mg/day PO) is an effective and safe option for managing DPN, as evidenced by improvements in TSS, NDS, and GSS. No significant improvements were observed in secondary outcomes, including VAS, VPT, NIS-LL, and NCS results.	Oral ALA treatment was generally safe and well-tolerated. Higher doses (above 600 mg daily) were associated with increased AEs, suggesting a dose-dependent safety profile. The most common side effects reported were mild GIT disturbances, such as nausea and vomiting.
Orellana-Donoso et al. [215]	A systematic review and meta-analysis evaluating the effectiveness of ALA in improving functional and symptomatic outcomes in patients with T1/T2 DM.	6 RCTs (n = 1077)	Compared to PLA, ALA did not exhibit significant differences in terms of pain reduction and various functional scales.	ALA was generally well-tolerated. The study did not report any severe AEs or significant safety concerns associated with the use of ALA for DPN.
Das et al. [216]	A systematic review and meta-analysis evaluating the efficacy and safety of oral ALA in managing DPN.	8 RCTs (n = 1797)	Oral 600 mg/day ALA (for up to 24 months) is an effective option for managing diabetic neuropathy.	At 600 mg/day, ALA was well-tolerated with minimal AEs. Higher doses (above 600 mg daily when given for ≥5 weeks) were associated with increased adverse effects, suggesting a dose-dependent safety profile.
Prado et al. [217]	A systematic review and meta-analysis to evaluate the efficacy and safety of oral ALA and GLA in managing DPN.	11 RCTs 9 RCTs using ALA (n = 1950)	9 RCTs: Oral 600 mg/day ALA is an effective and safe option for managing DPN. A dose-dependent response was observed, with higher doses correlating with greater symptom relief. No significant improvements were noted in secondary outcomes such as VPT, NIS-LL, and NCS results.	ALA treatment was generally well-tolerated, with mild GIT disturbances being the most common AEs.
Baicus et al. [22]	A systematic Cochrane review on the effectiveness of ALA in DPN	3 RCTs (n = 1262)	ALA, compared with PLA, has little or no effect on TSS and NIS-LL. A significant benefit cannot be ruled out because the lower 95% CI limit surpasses the MCID by 2 points.	There is minimal or no distinction between ALA and PLA regarding adverse events that result in treatment discontinuation within six months.

Abbreviations: AEs, adverse events; ALA, alpha-lipoic acid; DPN, diabetic peripheral neuropathy; GIT, gastrointestinal; GLA, gamma linolenic acid; GSS, global satisfaction score; IV, intravenous; MCID, minimal clinically important differences; NCS, nerve conduction study; NDS, neuropathy disability score; NIS-LL, Neuropathy Impairment Score for the Lower Limb; PLA, placebo; PO, per oral; RCT, randomized controlled trials; SOE, strength of evidence; TEAE, treatment-emergent adverse event; VAS, visual analogue scale; VPT, Vibration Perception Threshold.
